# Three-Year CD4/CD8 Ratio Recovery After Initiation of Dual Versus Triple Integrase Inhibitor–Based Therapy in Naïve Adults With HIV

**DOI:** 10.1093/ofid/ofag374

**Published:** 2026-06-20

**Authors:** Roser Navarro-Soler, Alejandro G García-Ruiz de Morales, Juan Macías, Jorge Sánchez-Villegas, José Luis Blanco Arévalo, Dàcil García Rosado, Antonio Ocampo Hermida, Cristina Gómez Ayerbe, Santiago Moreno, Javier Martínez-Sanz, Sergio Serrano-Villar

**Affiliations:** Department of Infectious Diseases, Hospital Ramón y Cajal, IRYCIS, Madrid, Spain; CIBERINFEC, Carlos III Health Institute, Madrid, Spain; Department of Infectious Diseases, Hospital Ramón y Cajal, IRYCIS, Madrid, Spain; CIBERINFEC, Carlos III Health Institute, Madrid, Spain; CIBERINFEC, Carlos III Health Institute, Madrid, Spain; Infectious Diseases and Microbiology Unit, Hospital Universitario Virgen de Valme, IBiS, Universidad de Sevilla, Sevilla, Spain; Clinical Unit for Infectious Diseases and Microbiology, Hospital Universitario Virgen de la Macarena IBiS, CSIC, Universidad de Sevilla, Sevilla, Spain; CIBERINFEC, Carlos III Health Institute, Madrid, Spain; HIV Unit, Hospital Clínic de Barcelona, Barcelona, Spain; Infectious Diseases Section, Internal Medicine Department, Hospital Universitario de Canarias, Canarias, Spain; Internal Medicine Department, Hospital Álvaro Cunqueiro, Galicia, Spain; Infectious Diseases Unit, Hospital Universitario Virgen de la Victoria, IBIMA-Plataforma BIONAND, Málaga, Spain; Department of Infectious Diseases, Hospital Ramón y Cajal, IRYCIS, Madrid, Spain; CIBERINFEC, Carlos III Health Institute, Madrid, Spain; Department of Medicine, Universidad de Alcalá, Madrid, Spain; Department of Infectious Diseases, Hospital Ramón y Cajal, IRYCIS, Madrid, Spain; CIBERINFEC, Carlos III Health Institute, Madrid, Spain; Department of Infectious Diseases, Hospital Ramón y Cajal, IRYCIS, Madrid, Spain; CIBERINFEC, Carlos III Health Institute, Madrid, Spain; Department of Medicine, Universidad Antonio de Nebrija, Madrid, Spain

**Keywords:** CD4/CD8 ratio, HIV, integrase inhibitors; 2-drug regimen; 3-drug regimen

## Abstract

From 21 233 adults in the Spanish HIV Research Network, we used propensity score matching to compare CD4/CD8 ratio recovery between dolutegravir-based 2DR and second-generation integrase inhibitor–based 3DR. No detectable differences in CD4/CD8 normalization were observed at 3 years across all cutoffs, suggesting that immune recovery does not differ between strategies.

Two-drug regimens (2DR) based on dolutegravir plus lamivudine (DTG + 3TC) have demonstrated non-inferior virological efficacy compared to 3-drug regimens (3DR) in randomized clinical trials [1, 2], leading to their endorsement as a first-line option in international guidelines [3]. Recent studies have progressively addressed initial concerns, extending efficacy and safety data to populations with low CD4 counts and settings without baseline resistance testing [4, 5]. However, long-term immunological recovery data in real-world settings remains limited.

In this context, the CD4/CD8 ratio is increasingly recognized as a clinically meaningful marker of immune health during HIV treatment. Unlike CD4 counts alone, the ratio reflects persistent immune activation and immunosenescence and has been independently associated with non-AIDS comorbidities and mortality in virologically suppressed individuals [6], with ratio normalization (>1) considered a marker of successful immune reconstitution [7]. Therefore, understanding how different treatment strategies affect CD4/CD8 dynamics in real-world settings is relevant for optimizing long-term outcomes.

Previous studies have consistently shown that integrase strand inhibitor (INSTI)–based regimens lead to greater CD4/CD8 ratio improvements compared to other drug classes [8]. However, most evidence comparing 2DR versus 3DR therapy derives from switch studies or short-term analyses. Our prior 48-week evaluation found no differences between strategies, but longer follow-up was needed to draw firm conclusions [9]. We therefore extended observation to 3 years to determine whether initiating ART with dolutegravir plus lamivudine results in comparable CD4/CD8 ratio recovery to INSTI-based 3DR regimens.

## METHODS

### Study Design, Participants, and Setting

Using a target trial emulation framework, we compared CD4/CD8 ratio recovery between treatment-naïve adults initiating a 2DR with DTG + 3TC and those initiating an INSTI-based 3DR, including bictegravir plus emtricitabine and tenofovir alafenamide (BIC/FTC/TAF) or dolutegravir combined with 2 NRTIs (3TC and abacavir, tenofovir disoproxil [TDF/FTC, or TAF/FTC]). Three outcomes were assessed: CD4/CD8 ratio normalization at 3 years using predefined cutoffs of 0.4, 1.0, and 1.5, spanning from the range associated with non-AIDS morbidity and mortality to full ratio normalization [10, 11].

We analyzed data from 21 233 treatment-naïve participants enrolled in the CoRIS Spanish Cohort [12]. Eligible individuals initiated ART between 30 June 2015 (first DTG/3TC use in CoRIS) and 30 November 2024. For each outcome, participants with a baseline CD4/CD8 ratio above the corresponding cutoff were excluded; each cutoff therefore defines a separate analytic cohort with a different baseline immunological profile (Supplementary Figure 1). Follow-up was censored at treatment modification or at 156 ± 12 weeks after ART initiation. Virologic failure was defined as 2 consecutive HIV-RNA measurements > 50 copies/mL after suppression, and a viral blip was defined as an isolated HIV-RNA measurement > 50 copies/mL after initial suppression, not followed by virologic failure.

### Statistical Analysis

We matched each participant who started 2DR with up to 2 participants who received 3DR using nearest-neighbor propensity score matching with a caliper of 0.05. Matching variables, selected according to prior knowledge [13], included age at cohort entry (within a 5-year range), sex, transmission category, educational level, AIDS diagnosis, baseline HIV RNA (log_10_ copies/mL), and nadir CD4 count. All variables included in the propensity score model were measured at or before ART initiation; no post-baseline variables were incorporated in the matching procedure. Subsequently, we fitted generalized estimating equation (GEE) models to allow for clustered data analysis (participants with 2DR and their matches) to assess the risk of ratio normalization at 3 years for each cutoff. Time to first achievement of each CD4/CD8 threshold was estimated with the Kaplan–Meier method and compared between groups using the log-rank test. Although the CD4/CD8 ratio is a repeatedly measured biomarker, the outcome of interest is the time to first achievement of each threshold, which is a single, non-recurrent event per participant per analysis. Covariate balance after matching was assessed using standardized mean differences (Supplementary Figure 2), and propensity score overlap between groups was evaluated graphically (Supplementary Figure 3). All statistical analyses were performed using Stata v. 18.0 (StataCorp LP College Station, TX, United States).

### Ethics

The Institutional Review Board of the Carlos III Health Institute located in Madrid and the Ethics Committee at University Hospital Ramón y Cajal approved the study. All participants provided their written informed consent to enter the CoRIS cohort. The CoRIS cohort was approved by the Research Ethic Committee of the Gregorio Marañón Hospital.

## RESULTS

From 6313 individuals who initiated bictegravir- or dolutegravir-based ART after 30 June 2015, 1139 started DTG/3TC. Table 1 summarizes the characteristics of the matched participants with a minimum follow-up of 39 months included in the analyses for CD4/CD8 cutoffs of 0.4, 1.0, and 1.5 (470, 788, and 849 participants, respectively). Covariate balance after matching was adequate across all models (standardized mean differences < 10%). The study population was predominantly young (median age 36 years), male (89%), and men who have sex with men (74%). Supplementary Table 1A and B present baseline and follow-up characteristics of participants before matching, respectively. Those initiating 3DR had higher rates of viral blips (17.8% vs 7.8%, *P* < .001) and virologic failure (5.0% vs 1.0%, *P* < .001), as well as lower baseline CD4 counts and CD4/CD8 ratios.

**Table 1.** Baseline Characteristics of Matched Participants, by CD4/CD8 Normalization Cutoff and Treatment Group

	CD4/CD8 ≥ 0.4		CD4/CD8 ≥ 1.0		CD4/CD8 ≥ 1.5	
	2DR, n = 173	3DR, n = 297	2DR, n = 307	3DR, n = 481	2DR, n = 325	3DR, n = 524
Age (years), median (IQR)	34.6 (28.4–43.8)	35.3 (28.5–42.2)	34.0 (27.9–42.4)	34.7 (28.2–42.6)	34.0 (27.9–42.2)	34.6 (27.7–43.3)
Gender, n (%)						
Male	162 (93.6)	279 (93.9)	289 (94.1)	453 (94.2)	305 (93.8)	496 (94.7)
Female	11 (6.4)	18 (6.1)	18 (5.9)	28 (5.8)	20 (6.2)	28 (5.3)
Mode of transmission, n (%)						
MSM	132 (76.3)	229 (77.1)	247 (80.5)	381 (79.2)	268 (82.5)	423 (80.7)
IDU	4 (2.3)	4 (1.3)	6 (2.0)	6 (1.2)	6 (1.8)	7 (1.3)
Heterosexual	32 (18.5)	54 (18.2)	47 (15.3)	82 (17.0)	50 (15.4)	91 (17.4)
Other	5 (2.9)	10 (3.4)	7 (2.3)	12 (2.5)	1 (0.3)	3 (0.6)
Country of origin, n (%)						
Spain	82 (47.4)	147 (49.5)	153 (49.8)	270 (56.1)	161 (49.5)	287 (54.8)
Western Europe	5 (2.9)	40 (13.5)	16 (5.2)	54 (11.2)	17 (5.2)	65 (12.4)
Eastern Europe	1 (0.6)	7 (2.4)	3 (1.0)	10 (2.1)	3 (0.9)	7 (1.3)
Sub-Saharan Africa	2 (1.2)	4 (1.3)	3 (1.0)	10 (2.1)	4 (1.2)	7 (1.3)
Northern Africa	6 (3.5)	1 (0.3)	6 (2.0)	2 (0.4)	7 (2.2)	4 (0.8)
Latin America	73 (42.2)	97 (32.7)	120 (39.1)	130 (27.0)	127 (39.1)	150 (28.6)
Other	4 (2.4)	1 (0.3)	6 (2.0)	5 (1.0)	6 (1.8)	4 (0.8)
Education level, n (%)						
None/incomplete primary	3 (1.7)	6 (2.0)	3 (1.0)	3 (0.6)	3 (0.9)	5 (1.0)
Primary	13 (7.5)	29 (9.8)	22 (7.2)	35 (7.3)	24 (7.4)	37 (7.1)
Compulsory secondary	20 (11.6)	33 (11.1)	34 (11.1)	58 (12.1)	36 (11.1)	83 (15.8)
High school	59 (34.1)	98 (33.0)	110 (35.8)	172 (35.8)	116 (35.7)	177 (33.8)
University or higher	56 (32.4)	93 (31.3)	103 (33.6)	155 (32.2)	112 (34.5)	157 (30.0)
Other	22 (12.7)	38 (12.8)	35 (11.4)	58 (12.0)	34 (10.5)	65 (12.4)
AIDS diagnosis at baseline, n (%)	0 (0.0)	0 (0.0)	0 (0.0)	0 (0.0)	0 (0.0)	0 (0.0)
Nadir CD4 (cells/µL), median (IQR)	387 (292–484)	339 (235–527)	415 (313–548)	419 (277–551)	433 (321–580)	415 (289–587)
Baseline HIV-RNA (log10), median (IQR)	4.8 (4.3–5.3)	4.9 (4.3–5.3)	4.7 (4.2–5.2)	4.8 (4.3–5.3)	4.7 (4.1–5.2)	4.7 (4.1–5.3)
Follow-up (years), median (IQR)	4.3 (3.8–5.1)	6.1 (4.7–7.8)	4.4 (3.8–5.2)	6.3 (4.8–7.9)	4.4 (3.8–5.1)	6.2 (4.7–7.7)

Participants matched according to age at cohort entry (within a 5-year range), sex, transmission category, educational level, baseline HIV RNA (log10), baseline nadir CD4, and AIDS diagnosis. For each outcome, we excluded participants who initiated ART with a CD4/CD8 ratio above the cutoff point. All comparisons within the same cutoff point yielded *P* > 0.05.

Abbreviations: ART, antiretroviral therapy; IDU, injecting drug use; MSM, men who have sex with men; 2DR: 2-drug regimen; 3DR: 3-drug regimen.

**Figure 1.**
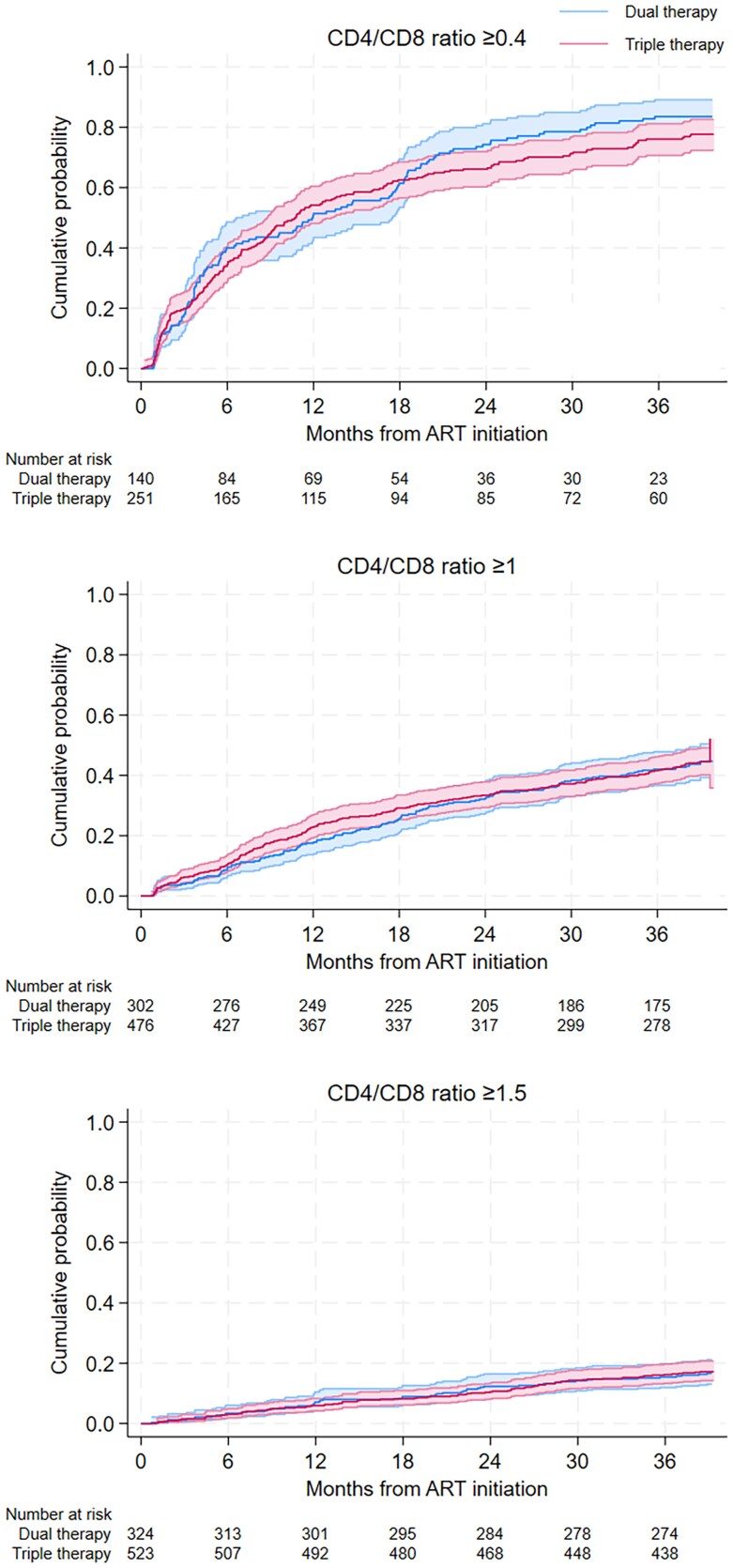
Kaplan–Meier survival estimates for CD4/CD8 ratio normalization at 0.4, 1.0, and 1.5 cutoffs in participants with dual and triple ART. The graph shows survival estimates for CD4/CD8 normalization for the 0.4 (*A*), 1.0 (*B*), and 1.5 (*C*) cutoff points, during 3 years of follow-up. Log-rank test *P*-values: *P* = .317 (≥0.4), *P* = .808 (≥1.0), and *P* = .869 (≥1.5).

At 3 years, 83% of participants achieved a CD4/CD8 ratio > 0.4, 45% achieved a ratio > 1.0, and 17% achieved a ratio > 1.5 (Figure 1). After matching according to the covariates of interest, GEE models showed no statistically significant differences in the probability of reaching a CD4/CD8 ratio ≥ 0.4 (OR 0.64, 95% CI .37–1.10), CD4/CD8 ≥ 1.0 (OR 0.99, 95% CI .75–1.31), or CD4/CD8 ≥ 1.5 (OR 1.03, 95% CI .71–1.49) between both treatment strategies (Supplementary Table 2). Figure 1 shows Kaplan–Meier survival plots of estimated overall CD4/CD8 normalization at cutoffs of 0.4, 1.0, and 1.5. There were no differences between 2DR and 3DR in the incidence rate of CD4/CD8 ratio normalization at 0.4, 1.0, and 1.5 cutoffs (Supplementary Table 3).

## DISCUSSION

In this large, nationwide cohort emulating a target trial, we found that antiretroviral therapy (ART) initiation with DTG + 3TC was not associated with detectable differences in the probability and timing of CD4/CD8 ratio normalization compared with BIC or DTG based-3DR over a 3-year period. These findings extend our previous observations at 48 weeks [9] and provide clinically relevant evidence that, in the medium term, immune recovery as measured by the CD4/CD8 ratio does not differ according to whether treatment is initiated with an INSTI-based 2DR or 3DR.

The CD4/CD8 ratio has emerged as a marker of persistent immune dysfunction in PWH on suppressive ART and is independently associated with non-AIDS comorbidities and mortality, even among individuals with adequate CD4 recovery [11]. Unlike CD4 counts alone, it captures broader dimensions of immune health related to immune activation and immunosenescence [6, 10], and the 3 thresholds used in this study reflect complementary stages of this recovery, from moving out of the range most strongly linked to non-AIDS events (≥0.4), through conventional normalization (≥1.0), to full recovery approaching the general-population median (≥1.5) [8, 10, 11]. Accumulating evidence indicates that the class of antiretroviral drugs, rather than the number of agents, plays a central role in shaping CD4/CD8 dynamics, with INSTI-based regimens showing faster improvement compared with non-nucleoside reverse transcriptase inhibitor (NNRTI)– or protease inhibitor–based therapies [13, 14]. This benefit is likely driven primarily by the rapid achievement of viral suppression characteristic of INSTI-based regimens, though the relative contribution of direct immunomodulatory effects remains to be established. However, long-term comparisons between INSTI-based 2DR and 3DR have been limited.

Our findings address this gap by extending follow-up to 3 years, allowing evaluation of CD4/CD8 trajectories beyond early immune reconstitution, when residual inflammation evolves more slowly [10, 15]. While our previous 48-week analysis already failed to detect differences [9], the persistence of similar CD4/CD8 normalization at 3 years suggests that the number of drugs does not influence medium-term immune recovery within INSTI-based regimens. These results align with randomized trials showing comparable virological efficacy and CD4 recovery with DTG/3TC versus 3DR [1, 2] and a recent observational European cohort reporting similar immunological trajectories [16].

Patients initiating bictegravir-based 3DR tend to have worse baseline immunovirological profiles and higher inflammatory burden [17], consistent with the higher rates of viral blips and lower baseline CD4 counts observed in our cohort. After matching, however, both strategies showed comparable CD4/CD8 recovery, indicating similar trajectories of immune reconstitution. Likewise, studies of residual viremia, total HIV-1 DNA, and inflammation have shown no differences between INSTI-based 2DR and 3DR [18–20]. Together with evidence that earlier treatment initiation improves CD4/CD8 normalization [21], these findings support the concept that immune recovery is driven primarily by INSTI use and treatment timing rather than regimen complexity, although our design does not allow comparison between individual 3DR combinations.

Current guidelines endorse dolutegravir plus lamivudine as first-line treatment [3], and our results provide immunological reassurance supporting this recommendation. Recent trial evidence has further extended the population in whom 2DR can be considered: the DOLCE trial demonstrated non-inferior efficacy in treatment-naïve individuals with baseline CD4 < 200 cells/µL [5], and D2ARLING confirmed efficacy in settings without baseline resistance testing [4]. Our findings add complementary real-world evidence, although participants with advanced immunosuppression remain under-represented in our cohort.

Several limitations should be considered. As an observational study without a prespecified equivalence framework, residual confounding cannot be entirely excluded despite rigorous target trial emulation and matching, and our results should be interpreted as no detected difference between strategies rather than as evidence of immunological equivalence. Additionally, although the CD4/CD8 ratio is a well-established surrogate marker of immune recovery, we did not directly assess clinical endpoints, such as non-AIDS events. Furthermore, the cohort was predominantly young, male, and MSM, which limits the generalizability of our findings to women, older adults, and other key populations. Nevertheless, the strong and consistent association between CD4/CD8 dynamics and long-term clinical outcomes supports the relevance of our findings. Strengths of this study include the large sample size, the use of a nationwide well audited cohort, the evaluation of multiple clinically relevant CD4/CD8 thresholds, and the extended follow-up duration compared with previous analyses. Future studies should link CD4/CD8 trajectories to clinical endpoints, such as non-AIDS events and mortality, and integrate the ratio with inflammatory markers to refine risk stratification.

In conclusion, in a large real-world cohort of PWH initiating INSTI-based therapy, no difference in CD4/CD8 ratio recovery was observed between 2DR with dolutegravir and lamivudine and 3DR over 3 years. These findings suggest that, within second-generation INSTI-based regimens, the number of antiretroviral agents does not appear to be associated with major differences in CD4/CD8 ratio recovery over 3 years.

## Supplementary Material

ofag374_Supplementary_Data

## Supplementary Data

Supplementary materials are available at *Open Forum Infectious Diseases* online. Consisting of data provided by the authors to benefit the reader, the posted materials are not copyedited and are the sole responsibility of the authors, so questions or comments should be addressed to the corresponding author.
