# Supplementary material for: Three-Year CD4/CD8 Ratio Recovery After Initiation of Dual Versus Triple Integrase Inhibitor–Based Therapy in Naïve Adults With HIV
Source: Open Forum Infect Dis. 2026 Jun 20;13(7):ofag374. doi: 10.1093/ofid/ofag374 (PMC13329665; doi:10.1093/ofid/ofag374)
Supplement: ofag374_Supplementary_Data [file ofag374_supplementary_data.zip › ofag374_Supplementary_Data.docx]

**SUPPLEMENTARY MATERIAL**

**Table S1A. Baseline characteristics of participants before matching**

| **Variable** | **Level** | **2-drug regimen (2DR)**  **(n=1,139)** | **3-drug regimen**  **(3DR)**  **(n=5,174)** | **p** |
| --- | --- | --- | --- | --- |
| **Sex, n (%)** | Male | 92.0 | 88.4 | <0.001 |
|  | Female | 8.0 | 11.6 |  |
| **Country of origin, n (%)** | Spain | 45.3 | 49.1 | <0.001 |
|  | Western Europe | 4.3 | 11.0 |  |
|  | Eastern Europe | 1.7 | 2.0 |  |
|  | Sub-Saharan Africa | 1.2 | 2.7 |  |
|  | Northern Africa | 1.8 | 1.4 |  |
|  | Latin America | 44.2 | 32.4 |  |
|  | Other | 1.5 | 1.5 |  |
| **Education level, n (%)** | None / incomplete primary | 1.4 | 1.5 | <0.001 |
|  | Primary | 5.2 | 8.6 |  |
|  | Compulsory secondary | 12.5 | 12.7 |  |
|  | High school | 34.5 | 28.1 |  |
|  | University or higher | 31.4 | 26.5 |  |
|  | Other | 1.3 | 1.4 |  |
|  | Unknown | 13.7 | 21.0 |  |
| **Mode of HIV acquisition, n (%)** | MSM | 78.4 | 69.0 | <0.001 |
|  | IDU | 1.4 | 1.9 |  |
|  | Heterosexual | 16.6 | 23.4 |  |
|  | Other | 3.6 | 5.8 |  |
| **AIDS diagnosis at baseline, n (%)** | Yes | 2.2 | 12.2 | <0.001 |
| **Nadir CD4 (cells/µL), median (IQR)** |  | 437 (320–605) | 354 (199–518) | <0.001 |
| **Nadir CD4/CD8, median (IQR)** |  | 0.49 (0.33–0.69) | 0.36 (0.20–0.58) | <0.001 |
| **Baseline HIV-RNA (log10 copies/mL), median (IQR)** |  | 4.65 (4.08–5.21) | 5.03 (4.46–5.58) | <0.001 |

*Variables measured at or before the date of ART initiation. Percentages shown for categorical variables; median (IQR) for continuous variables.* *Abbreviations: 2DR, two-drug regimen; 3DR, three-drug regimen; IDU, injection drug use; IQR, interquartile range; MSM, men who have sex with men.*

**Table S1B. Follow-up characteristics of participants before matching**

| **Variable** | **Level** | **2-drug regimen (2DR)**  **(n=1,139)** | **3-drug regimen**  **(3DR)**  **(n=5,174)** | **p** |
| --- | --- | --- | --- | --- |
| **Duration of 1st ART regimen (months), median (IQR)** |  | 22.2 (11.4–40.1) | 27.7 (9.9–49.4) | <0.001 |
| **Follow-up duration (months), median (IQR)** |  | 23.4 (8.6–43.0) | 43.1 (16.5–73.8) | <0.001 |
| **Time from ART initiation to VL<50 (weeks), median (IQR)** |  | 9 (4–21) | 13 (6–29) | <0.001 |
| **Number of VL determinations during follow-up, median (IQR)** |  | 5 (3–8) | 8 (4–13) | <0.001 |
| **Viral blip ever during follow-up, n (%)** | No | 92.2 | 82.2 | <0.001 |
|  | Yes | 7.8 | 17.8 |  |
| **Virologic failure ever during follow-up, n (%)** | No | 99.0 | 95.0 | <0.001 |
|  | Yes | 1.0 | 5.0 |  |
| **AIDS-related death during follow-up, n (%)** | Yes | 0.1 | 0.4 | 0.077 |

*Variables measured after ART initiation. Percentages shown for categorical variables; median (IQR) for continuous variables.Abbreviations: 2DR, two-drug regimen; 3DR, three-drug regimen; ART, antiretroviral therapy; IQR, interquartile range; VF, virologic failure; VL, viral load.*

*Viral blip: isolated HIV-RNA >50 copies/mL after initial suppression, not followed by virologic failure. Virologic failure: two consecutive HIV-RNA measurements >50 copies/mL after suppression.*

**Table S2. Generalized estimating equation (GEE) models for ratio normalization at 3 years**

| **ART group** | **OR** | **95% CI** | **p** |
| --- | --- | --- | --- |
| **CD4/CD8 ratio ≥0.4** | | | |
| 2-drug regimen (2DR) | Ref. | — | — |
| 3-drug regimen (3DR) | 0.64 | 0.37, 1.10 | 0.107 |
| **CD4/CD8 ratio ≥1.0** | | | |
| 2-drug regimen (2DR) | Ref. | — | — |
| 3-drug regimen (3DR) | 0.99 | 0.75, 1.31 | 0.939 |
| **CD4/CD8 ratio ≥1.5** | | | |
| 2-drug regimen (2DR) | Ref. | — | — |
| 3-drug regimen (3DR) | 1.03 | 0.71, 1.49 | 0.888 |

*Participants matched according to age at cohort entry (within a 5-year range), sex, transmission category, educational level, AIDS diagnosis, nadir CD4, and baseline HIV-RNA (log10 copies/mL), using nearest-neighbour matching (up to 2 neighbours) with a caliper of 0.05. Abbreviations: CI, confidence interval; OR, odds ratio; Ref., reference category.*

**Table S3. Incidence rates of CD4/CD8 normalization at different cutoff points**

|  | **Incidence rate (per 1000 person-years)** | **95% CI** |
| --- | --- | --- |
| **CD4/CD8 ratio ≥0.4** | | |
| 2-drug regimen (2DR) | 53 | 45, 64 |
| 3-drug regimen (3DR) | 46 | 40, 53 |
| **CD4/CD8 ratio ≥1.0** | | |
| 2-drug regimen (2DR) | 15 | 13, 18 |
| 3-drug regimen (3DR) | 16 | 14, 18 |
| **CD4/CD8 ratio ≥1.5** | | |
| 2-drug regimen (2DR) | 5 | 4, 6 |
| 3-drug regimen (3DR) | 5 | 4, 5 |

*Abbreviations: CI, confidence interval*

**Figure S1. Cohort flow diagram for each CD4/CD8 normalisation cut-off analysis.** The three cohorts are not nested: each cut-off analysis starts from the same population of 6,608 participants initiating INSTI-based ART and applies three sequential exclusions: (i) baseline CD4/CD8 ratio already above the corresponding cut-off; (ii) less than 39 months of follow-up; and (iii) no feasible match. Numbers within each box indicate total participants and distribution by treatment group (2DR, two-drug regimen; 3DR, three-drug regimen).

**Figure S2. Standardised mean differences of matching covariates before and after propensity score matching.** Absolute standardised bias for each covariate, before (dots) and after (crosses) matching, in the analytic cohorts for CD4/CD8 cut-offs ≥0.4 , ≥1.0 and ≥1.5. All standardised differences were below 10% after matching in the three cohorts.

**Figure S3. Propensity score distributions before and after matching.** Distribution of estimated propensity scores in the 2DR and 3DR groups, before and after matching, for the analytic cohorts of CD4/CD8 cut-offs ≥0.4 (A), ≥1.0 (B) and ≥1.5 (C). Adequate overlap and common support were observed in all three cohorts

**Supplementary_Appendix_CoRIS_Authors**

***CoRIS Executive committee:***

Santiago Moreno, Inma Jarrín, David Dalmau, M Luisa Navarro, Federico Garcia, Eva Poveda, Jose Antonio Iribarren, Félix Gutiérrez, Francesc Vidal, Juan Berenguer, Juan González.

***Centres and investigators involved in CoRIS cohort are listed below:***

**CoRIS Coordination Unit**

Inma Jarrín, Cristina Moreno, Marta Rava, Rebeca Izquierdo, Cristina Marco-Sánchez, Teresa Gómez-García.

**BioBanK HIV Hospital General Universitario Gregorio Marañón**

José Luis Jiménez.

**Hospital General Universitario Dr Balmis (Alicante)**

Sergio Reus, Irene Portilla, Esperanza Merino, Gema García, José Sánchez-Payá, Juan Carlos Rodríguez, Livia Giner, Joaquín Portilla, Vicente Boix, Diego Torrus, Julia Portilla-Tamarit, Héctor Pinargote.

**Hospital Universitario de Canarias (San Cristóbal de la Laguna)**

María Remedios Alemán, Nereyda Tosco-García, Ana López Lirola, Dácil García, Felicitas Díaz-Flores, María del Mar Alonso, Ricardo Pelazas, María Inmaculada Hernández, Lucia Romero-Acevedo, Abraham Bethencourt-Padilla, Daniel Rodríguez-Díaz, Ana María Godoy-Reyes.

**Hospital Universitario Central de Asturias (Oviedo)**

Víctor Asensi, Rebeca Cabo Magadan, Lorena Fernández, Javier Díaz-Arias

**Hospital Universitario 12 de Octubre (Madrid)**

Federico Pulido, Rafael Rubio, M Asunción Hernando, Otilia Bisbal, David Rial-Crestelo, María de Lagarde, Laura Bermejo, Mireia Santacreu, Juan Martín Torres, Belén Sánchez-López.

**Servicio de Enfermedades Infecciosas. Hospital Universitario Donostia. Instituto de Investigación Sanitaria BioGipuzkoa (Donostia-San Sebastián)**

José Antonio Iribarren, Xabier Kortajarena, Claudia Nevado Pavón, Xabier Camino, Miguel Ángel Goenaga, M Jesús Bustinduy, Harkaitz Azkune, Maialen Ibarguren, Ignacio Álvarez-Rodriguez, Leire Gil-Alonso, Francisco Carmona-Torre, Ana Bayona Carlos, Maialen Lekuona Sanz, Leire Balerdi Sarasola, Ander Goyache Moreno.

**Hospital General Universitario De Elche (Elche)**

Félix Gutiérrez, Catalina Robledano, Mar Masiá, Sergio Padilla, Rafael Pascual, Marta Fernández, Antonio Galiana, José Alberto García, Xavier Barber, Javier García Abellán, Guillermo Telenti, Ángela Botella, Paula Mascarell, Lidia García-Sánchez, Nuria Ena, Leandro López, Jennifer Vallejo, Nieves Gonzalo-Jiménez, Montserrat Ruiz, Christian Ledesma, Santiago López, María Espinosa-Pérez, Ana Quiles, María del Mar Alcalde-Encinas, José García-García, Rosario Hernández-Ros, José Carlos Escribano, Marouane Menchi-Elanci, María del Mar García Navarro, Melissa Bello Pérez.

**Hospital General Universitario Gregorio Marañón (Madrid)**

Cristina Diez, Isabel Gutiérrez, Juan Berenguer, Margarita Ramírez, Teresa Aldamiz-Echevarría, Francisco Tejerina, Leire Pérez-Latorre, Chiara Fanciulli, Saray Corral.

**Hospital Universitari de Tarragona Joan XXIII (Tarragona)**

Joaquim Peraire, Anna Rull, Anna Martí, Consuelo Viladés, Beatriz Villar, Lluïsa Guillem, Silvia Chafino, Marina Flores, Francesc Vidal.

**Hospital Universitario y Politécnico de La Fe (Valencia)**

Marta Montero-Alonso, María Tasias-Pitarch, Eva Calabuig-Muñoz, Miguel Salavert-Lletí, Juan Fernández-Navarro, Rosa Blanes-Hernández, Jennifer Sánchez-Guevara.

**Hospital Universitario La Paz/IdiPAZ (Madrid)**

Juan González-García, José Ignacio Bernardino, Ana Delgado-Hierro, José Ramón Arribas, Víctor Arribas, Juan Miguel Castro, Luis Escosa, Iker Falces, Pedro Herranz-Pinto, Alicia González-Baeza, María Luz Martín-Carbonero, Rafael Micán, Rocío Montejano, María Luisa Montes, Luis Ramos-Ruperto, Berta Rodés Soldevila, Talia Sainz, Elena Sendagorta, Carmen Busca, Joanna Cano-Smith, Rosa de Miguel, María del Mar Arcos-Rueda, Alejandro de Gea-Grela, Nerea Iniesta-Arandia, Alejandro Díez-Vidal, María Jesús Roldán-Cabrales, Carlos Oñoro-López, Jara Llenas García, Laura Lucía Checa Daimiel.

**Hospital Universitari Mutua Terrassa (Terrassa)**

David Dalmau, Marina Martinez, Angels Jaén, Mireia Cairó, Javier Martinez-Lacasa, Roser Font, Laura Gisbert.

**Hospital Universitario de La Princesa (Madrid)**

Ignacio de los Santos, Alejandro de los Santos, Lucio García-Fraile, Enrique Martín-Gayo, Ildefonso Sánchez-Cerrillo, Ángela Gutiérrez, Carmen Sáez, Ana Barrios Blandino, Azucena Bautista, Marianela Ciudad, María Aguilera García, Violeta Sampériz Rubio, Javier Pérez Serrano, Isabel Belmonte Martín de Santa Olalla.

**Hospital Universitario Ramón y Cajal (Madrid)**

Santiago Moreno, Santos del Campo, José Luis Casado, Fernando Dronda, Ana Moreno, María Jesús Pérez-Elías, Sergio Serrano-Villar, María Jesús Vivancos-Gallego, Javier Martínez-Sanz, Alejandro Vallejo Tiller, Matilde Sánchez-Conde, José Antonio Pérez-Molina, José Manuel Hermida, Erick De La Torre Tarazona, Elena Moreno del Olmo, Laura Martín Pedraza, Claudio Díaz García, Jorge Díaz Álvarez, Alejandro García-García, Raquel Ron-González, Sergio Calderón Vicente, Roser Navarro-Soler, Sara Saiz-Baggetto, Ana del Amo-de Palacios, Laura Luna, Miguel Antón Ámez Segovia, Sara Martín Colmenarejo, Cristina Chica.

**Hospital General Universitario Reina Sofía (Murcia)**

Enrique Bernal, María Dolores Hernández, Antonia Alcaraz, Joaquín Bravo, Ángeles Muñoz Pérez, Cristina Tomás Jimenez, Salvador Valero Cifuentes, Eva García-Villalba, Román González Hipólito, Elena Guijarro-Westermeyer, Rodrigo Martínez-Rodríguez, José Miguel Gómez Verdú.

**Hospital Universitario Clínico San Cecilio (Granada)**

Federico García, Clara Martínez, Maite Laperal, Leopoldo Muñoz Medina, Marta Álvarez-Estevez, Natalia Chueca-Porcuna, David Vinuesa-García, Adolfo de Salazar-González, Ana Fuentes-López, Emilio Guirao, Andrés Ruiz-Sancho, Francisco Anguita, Naya Faro, Lucia Chaves, Marta Illescas, Paloma Muñoz-Baez, Lucía Pérez, Ana Alberola Romano, Alberto Vazquez Blanquiño, Lucía Guillén-Zafra, Javier Martínez de Victoria-Carazo.

**Centro Sanitario Sandoval (Madrid)**

Jorge Del Romero-Guerrero, Montserrat Raposo, Teresa Puerta-López, Mar Vera, Juan Ballesteros, Begoña Baza, Laura Dans Villán, Ruben Linares Navarro, Ines Armenteros Yeguas, Eva Orviz-García, Santiago Fernández Castelao.

**Hospital Universitario Son Espases (Palma de Mallorca)**

Melchor Riera, Antonio Vanrell, María Peñaranda, Mª Angels Ribas, Antoni A. Campins, Mercedes Garcia-Gasalla, Francisco J Fanjul, Javier Murillas-Angoiti, Luisa Martin-Pena, Francisca Artigues, Sophia Pinecki, Adrián Ferre.

**Hospital Universitario Virgen de la Victoria (Málaga)**

Jesús Santos, María López-Jódar, Cristina Gómez-Ayerbe, Isabel Viciana, Rosario Palacios.

**Hospital Universitario Virgen del Rocío (Sevilla)**

Luis Fernando López-Cortés, Silvia Llaves-Flores, Inmaculada Rivas Jeremías, Nuria Espinosa, Cristina Roca-Oporto, Marta Herreros-Romero, César Sotomayor de la Piedra, Abraham Saborido Alconchel, Manuel Francisco Liroa, Jesús Fernández Plaza.

**Hospital Universitario de Bellvitge (Hospitalet de Llobregat)**

Juan Manuel Tiraboschi, Arkaitz Imaz, María Saumoy, Analuz Fernandez, Jaime Vega Costa, Daniel Medina Gamito.

**Hospital Costa del Sol (Marbella)**

Julián Olalla, Javier Pérez, Alfonso del Arco, Javier de la Torre, Francisca Ruiz.

**Hospital General Universitario Santa Lucía (Cartagena)**

Onofre Juan Martínez, Lorena Martinez, Francisco Jesús Vera, Josefina García, Begoña Alcaraz, Sergio Guillén Martínez, Patricia Carles García.

**Complejo Hospitalario Universitario a Coruña (CHUAC) (A Coruña)**

Álvaro Mena, Berta Pernas, Pilar Vázquez, Soledad López, Brais Castelo.

**Hospital Universitario Virgen de la Arrixaca (El Palmar)**

Carlos Galera, Marian Fernández, Helena Albendin, Antonia Castillo, Asunción Iborra, Antonio Moreno, M Angustias Merlos, Almudena Ortuño.

**Hospital Universitario Infanta Sofía (San Sebastián de los Reyes)**

Inés Suarez-García, Eduardo Malmierca, Patricia González-Ruano, M Pilar Ruiz, Luz Balsalobre, Ángela Somodevilla, Rebeca Fuerte Martínez.

**Hospital Clínico San Carlos (Madrid)**

Vicente Estrada, Nieves Sanz, Noemí Cabello-Clotet, María José Núñez, Ana Muñoz, Juncal Pérez-Somarriba, Reynaldo Homen, Rafael Rubio-Martín, Susana Olmedo, Julia Barrado.

**Hospital Universitario Príncipe de Asturias (Alcalá de Henares)**

José Sanz, Cristina Hernández-Gutiérrez, María Novella-Mena.

**Hospital Clínico Universitario de Valencia (Valencia)**

María José Galindo, Sandra Pérez Gómez, Ana Ferrer, Anaís Corma Gómez.

**Hospital Reina Sofía (Córdoba)**

Antonio Rivero-Román, Laura Ruiz-Torres, Antonio Rivero-Juárez, Pedro López-López, Mario Frias-Casas, Ángela Camacho, Ignacio Pérez, Diana Corona, Javier Manuel Caballero, Marina Gallo Marín, María Casares, Lucía Ríos-Muñoz, Claudia Ferreira-Tata, María Carrasquilla.

**Hospital Universitario Severo Ochoa (Leganés)**

Rafael Rodríguez-Rosado Martinez-Echevarría.

**Hospital Universitario Virgen de Valme (Sevilla)**

Juan Macías Sánchez, Pilar Rincón, Luis Miguel Real, Jésica Martín-Carmona.

**Hospital Álvaro Cunqueiro (Vigo)**

Eva Poveda, Alexandre Pérez, Luis Morano, Celia Miralles, Antonio Ocampo, Jacobo Alonso, Inés Martínez, Aida López-López.

**Hospital Universitario Marqués de Valdecilla (Santander)**

M Carmen Fariñas Álvarez, Claudia González Rico, Noelia Ruiz Alonso, Carlos Armiñanzas Castillo, Francisco Arnaiz de las Revillas Almajano, Manuel Gutiérrez Cuadra, Raúl Parra Fariñas, Paula Runza Buznego, Aitziber Illaro Uranga.

**Hospital Clínic de Barcelona (Barcelona)**

José Luis Blanco Arévalo, Pilar Callau Cabrera, Josep Mallolas Masferrer, José Alcamí Pertejo, Sonsoles Sánchez-Palomino, Núria Climent, Ana González-Cordón, Montse Laguno Centeno, María Martínez-Rebollar, Juan Ambrosioni, Berta Torres, Lorena de la Mora, Alexy Inciarte, Esteban Martínez, José María Miró, Abiu Sempere, Julia Calvo, Iván Chivite, David García, Alberto Foncillas, Daniela Malano, Montse Plana, Roger Llobet, Estela Solbes, Octavi Roman, Rona Sagarra, Vanesa Guilera, Gemma Olmeda, Paula Arreba, María José Rodríguez, Emma Fernández, Ana Rodríguez, Alba ortega, Sergi Anguera, Raquel Aguiló, Laura Novell.

**Centro Nacional de Microbiología (Majadahonda)**

Luis Miguel Bedoya Del Olmo, Manuela Beltran Vicente, Mercedes Bermejo Herrero, Maria Esther Calonge Errejon, Laura Capa Muñoz, Maria Teresa Coiras Lopez, Francisco Diez Fuertes, Javier Garcia Perez, Nuria Gonzalez Fernandez, Elena Mateos De Las Moreras, Maria Teresa Perez Olmeda, Victor Sanchez Merino, Maria Eloisa Yuste Herranz.

**Centro Nacional de Biotecnología. Consejo Superior de Investigaciones Científicas (CSIC) (Madrid)**

Carmen Elena Gomez Rodriguez, Juan Francisco Garcia Arriaza, Laura Sin Diaz, Beatriz Perdiguero de la Torre, Laura Marcos Villar, Patricia Perez Ramirez, Enrique Alvarez Coruña, David Astorgano Lopez, Cristina Sanchez Corzo.
