# Supplementary figures and images for: Three-Year CD4/CD8 Ratio Recovery After Initiation of Dual Versus Triple Integrase Inhibitor–Based Therapy in Naïve Adults With HIV
Source: Open Forum Infect Dis. 2026 Jun 20;13(7):ofag374. doi: 10.1093/ofid/ofag374 (PMC13329665; doi:10.1093/ofid/ofag374)

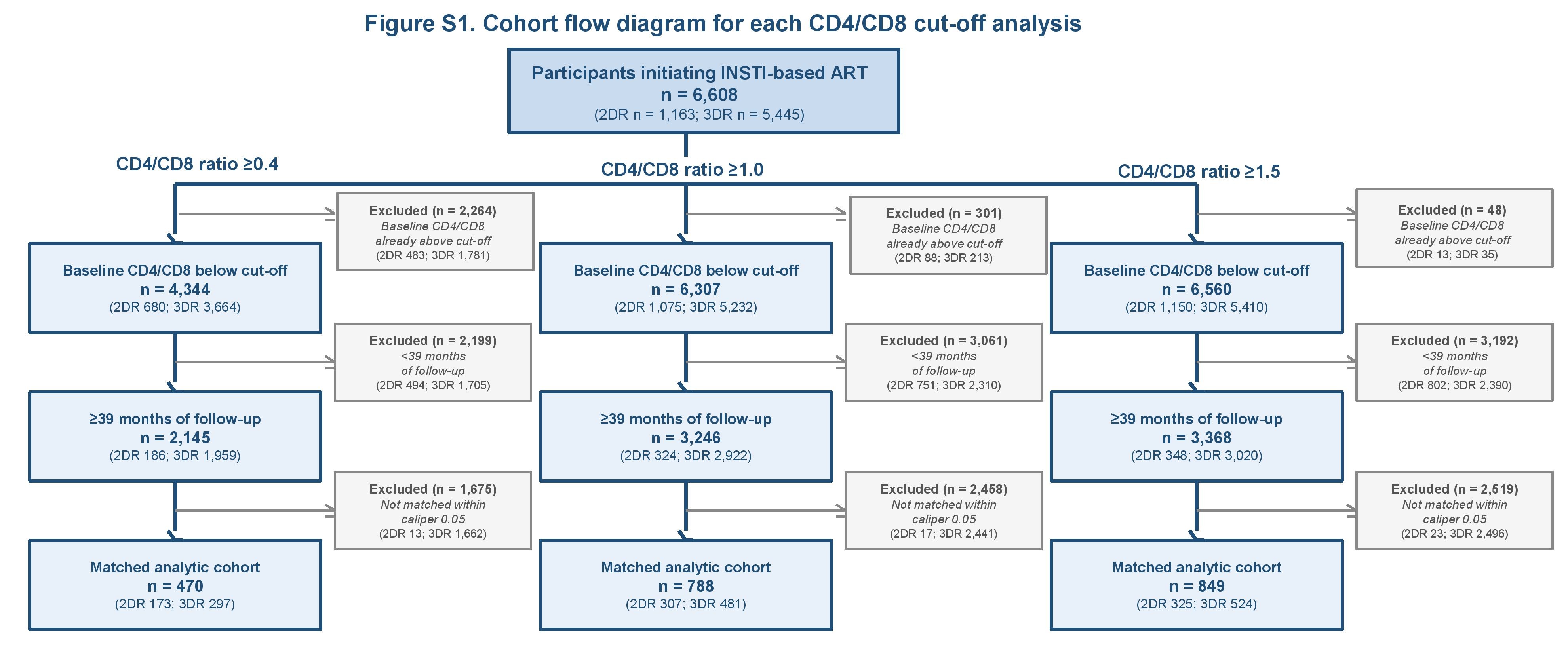

Supplement: ofag374_Supplementary_Data [file ofag374_supplementary_data.zip › Figure_S1_flow_diagram.jpg]

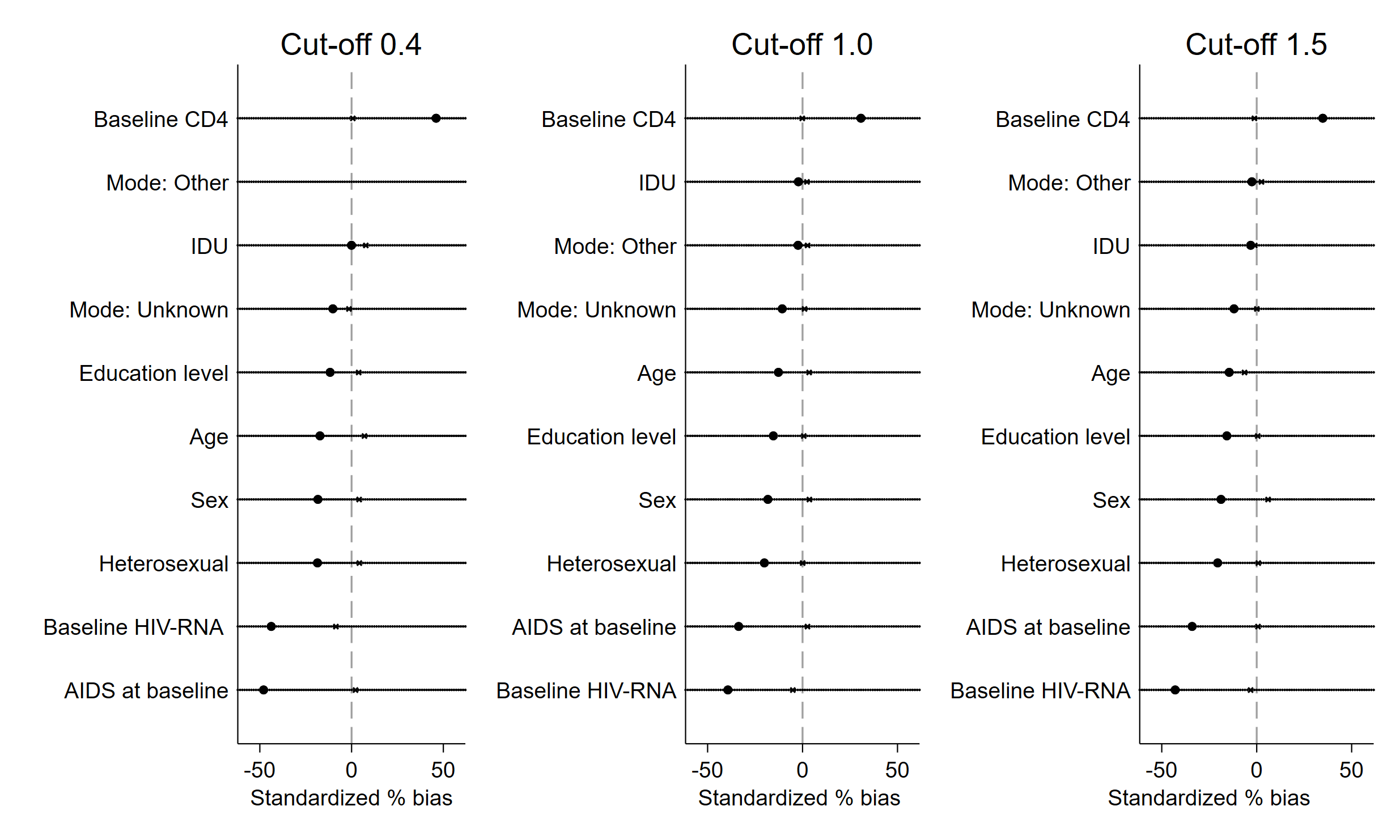

Supplement: ofag374_Supplementary_Data [file ofag374_supplementary_data.zip › Figure_S2.tif]

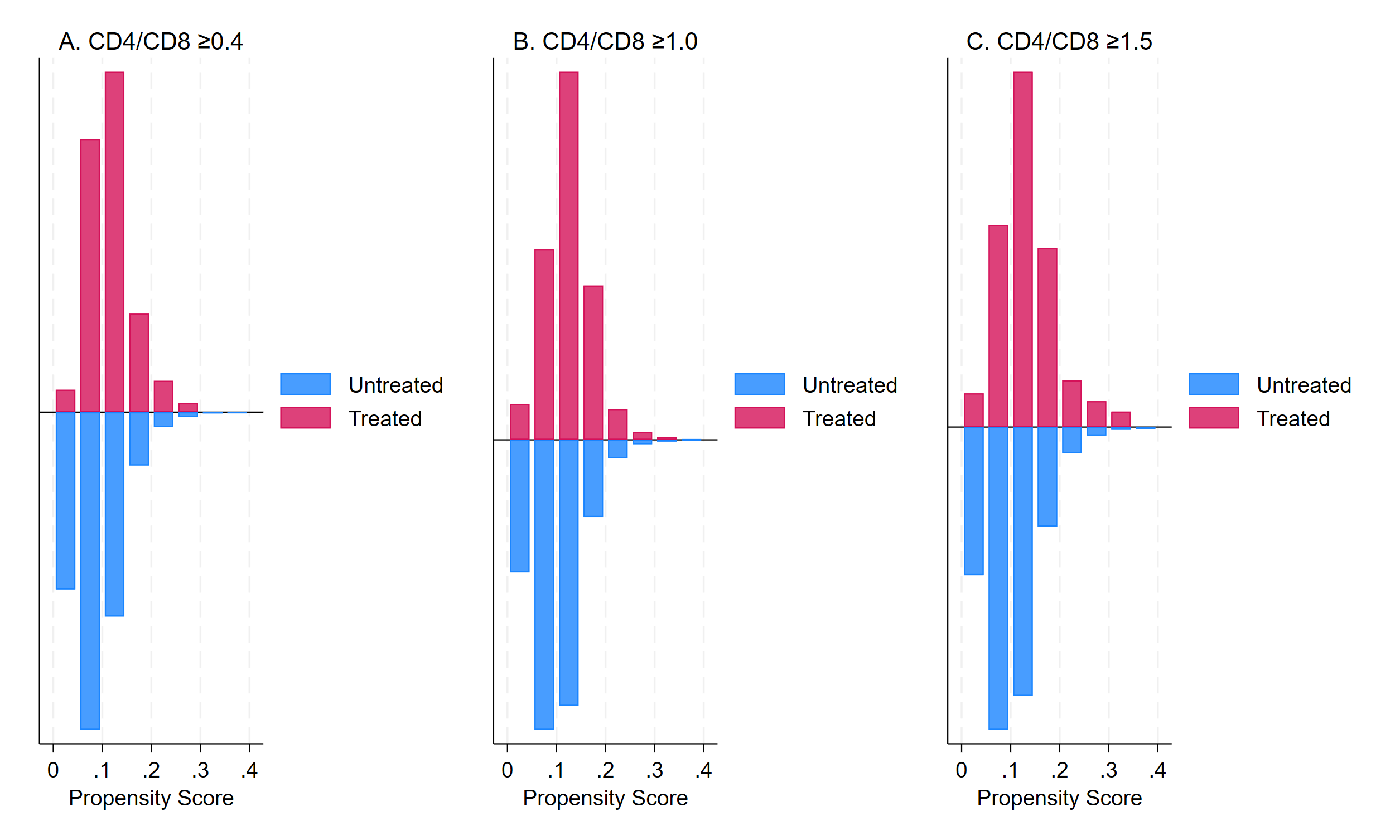

Supplement: ofag374_Supplementary_Data [file ofag374_supplementary_data.zip › Figure_S3.tif]
